# Morphometrics of waterlogged archaeological seeds give new insights into the domestication and spread of *Papaver somniferum* L. in Western Europe

**DOI:** 10.1371/journal.pone.0286190

**Published:** 2023-05-25

**Authors:** Ana Jesus, Vincent Bonhomme, Allowen Evin, Raül Soteras, Stefanie Jacomet, Laurent Bouby, Ferran Antolín

**Affiliations:** 1 Department of Environmental Sciences, Integrative Prehistory and Archeological Science (IPAS), University of Basel, Basel, Switzerland; 2 Département Paléo-Ecosystèmes, Climat, Sociétés (PAST), ISEM, University of Montpellier-CNRS-IRD-EPHE, Montpellier, France; 3 Division of Natural Sciences, German Archaeological Institute, Berlin, Germany; University of California Santa Cruz, UNITED STATES

## Abstract

Domesticated opium poppy *Papaver somniferum* L. subsp. *somniferum* probably originated in the Western Mediterranean from its possible wild progenitor, *Papaver somniferum* L. subsp. *setigerum* and spread to other European regions. Seeds of opium poppy have been identified in different European regions since the Early Neolithic (from the 6^th^ millennium cal. BC onwards) period. However, until recently, the absence of morphological identification criteria has prevented the discrimination between wild and domestic morphotypes. New morphometric approaches to distinguish modern subspecies have been proven to be applicable to waterlogged archaeological remains, opening the possibility of understanding the process of domestication of the plant in both time and space. This paper applies seed outline analyses, namely elliptic Fourier transforms, combined with size and number of cells to archaeological waterlogged *Papaver* seeds throughout the Neolithic period in the NW Mediterranean and the surroundings of the Alps. Furthermore, one example from the Late Bronze Age (LBA) was added to see what kind of differences appeared during the >1000 years between the end of the Neolithic and the LBA. The aim of the study is to classify the archaeological seeds as domestic or wild morphotypes and observe morphometric changes in connection to geographical and chronological patterns that can explain the spread and domestication process(es) of this important crop. A total of 295 archaeological seeds coming from 10 waterlogged sites dating between 5300–2300 cal. BC (Neolithic), and one LBA site dating to 1070 cal. BC were analysed. The results indicate the presence of seeds, similar to the wild morphotype, in the Mediterranean sites and larger seeds, similar to the domestic morphotype, in the regions surrounding the Alps. The number of cells mainly increased during the Late Neolithic (3300 to 2300 cal. BC) and, finally, in the Late Bronze Age (ca. 1050–800 cal. BC), larger, morphologically domesticated seeds are clearly predominant. A change in the shape of the seeds is only clearly visible in the LBA material. Altogether our results suggest that opium poppy seeds show no sign of domestication in the early periods of the Neolithic, despite the fact that the plant was very probably already cultivated at that time in the western Mediterranean region.

## Introduction

Plant domestication in Southwest Asia and Europe has been approached by archaeobotanists through a variety of techniques, including seed size [1,2], the proportion of botanical macro remains presenting domestication traits [3,4] and, more recently, seed shape analysis [5,6]. This has led to different narratives on the pathways of domestication of different old crops [7] but the datasets still remain scarce due to preservation issues and often inconclusive due to the insufficient diagnostic traits. In addition, the domestication process is complex and researchers are only starting to grasp it. Among the limitations of these studies one could highlight the sole possibility of analysis of charred material, which is always a biased representation of past plant populations (especially of oil crops) and hence limits the availability of representative material [8]. A second limitation is the long time-span that exists between the establishment of a broad-spectrum economy amongst the last hunter-gatherers (and the beginning of a long co-evolutionary process with plants and animals that would end in some domestication events) and the development of agriculture (a necessary step in plant domestication). The domestication process in this sense was clearly human-driven but not deliberate from the beginning and hence difficult to trace [9]. Secondary domestication centres (where new plant species were domesticated once the societies became agricultural) [10] may offer the possibility of documenting domestication processes over shorter periods of time, since they do not run in parallel to the development of agricultural practices, which are already known to the society. Our case study is opium poppy (*Papaver somniferum* L. subsp. *somniferum*, further abbreviated as *P*. *somniferum*), a plant that could have been domesticated in Western Mediterranean Europe from a wild progenitor (*Papaver somniferum* (DC.) Corb. subsp. *setigerum*, further abbreviated as *P*. *setigerum*) after the arrival of the first farming populations [11–15]. The seeds of opium poppy are very well documented in, among other sites, lakeshore and bog settlements with excellent preservation conditions (waterlogged) that allow working with uncharred subfossil seeds whose size and shape remain much closer to the original than the carbonized seeds.

Currently, the largest producer of opium poppy is Afghanistan, with 6.000 tons of opium and 250.00 hectares cultivated per year [16]. The Czech Republic, France and Russia are also important producers [16]. The opium poppy is nowadays mainly used for its medicinal, alimentary and decorative purposes [17]. In the past, opium poppy might have been collected for its seeds, which are rich in fibre, healthy fats and several micronutrients such as manganese, copper and iron [18,19]. Despite its current and past economic importance, opium poppy domestication has been challenging to approach archaeologically. Most researchers convey that the domestication occurred or was initiated during the Neolithic period in Mediterranean Western Europe [12,13,15,20], making it the only plant known to have been domesticated in Europe during the Neolithic. This hypothesis, however, is far from being proven, because there are considerable genetic differences in both *Papaver* subspecies, *P*. *somniferum* ssp. *setigerum* being mostly tetraploid whilst the cultivar is diploid [21,22]. Accessions of *P*. *setigerum*, however, are largely under-studied as stated in Hong et al. [21], and–as the authors state–there are also diploid forms of *P*. *setigerum* known, but not yet investigated genetically.

Domestication refers to genetic, morphological and physiological changes in plants and animals [23]. Pre-domestication cultivation refers to the continuous management of wild progenitors (planting, harvesting and storing) and soil preparation by land clearance and tillage; these practices reflect as well on the evolution of larger seeds and reduction of the dispersal aids [23]. The main domestication traits of poppy are the increase in the size of the capsules and seeds and the indehiscence of the capsule [11]. A rare finding of complete capsules of opium poppy is known from pictures of material (currently lost) from Murciélagos cave in Spain [24–26], presumably dating to the Late Neolithic period (3300–2300 BC cal. BC). Whole capsules are almost never preserved in archaeological sites (e.g. lid-parts were found uncharred in the Late Neolithic sites north of the Alps such as Zurich-Parkhaus Opera around 3100 cal. BC [27] and in charred state in a burnt layer (AH2) of Hornstaad Hörle IA site, dated to ca. 3910 cal. BC [28]). Therefore, only two options are available to study the domestication and spread of this species: seed size and geographic spread. The latter refers to the spread of the plant (evidenced by seed finds) beyond its native area and therefore a sign that it might have been cultivated, either as a weed or as a crop on its own.

In the archaeological record, the seeds are the most common remains of this plant, preserved charred in dryland or mostly uncharred in waterlogged environments. Unfortunately, they are usually found in very small amounts in dryland sites because of the bad survival chances of the oil rich seeds when exposed to heat, and their extreme fragility when preserved in a charred state. In addition to this, the seeds are tiny (<1mm length) and require adequate sampling and very gentle sieving (the so-called “wash-over” method sensu Kenward et al. [29,30]) with a small-sized mesh. Large seed numbers are almost exclusively known from wetland sites, where uncharred (waterlogged) seeds (and rare capsule fragments, mostly lid parts, see above) are usually preserved in higher numbers (for numbers of finds, see e.g. [31]). Therefore, it is uncertain to what extent taphonomy has played an important role in the reconstruction of opium poppy history (i.e. it could have been present in other regions and gone unnoticed).

The archaeological *P*. *somniferum* might correspond to three current subspecies. These are *P*. *setigerum*, the putative wild ancestor, and two varieties of the domestic subspecies, *Papaver somniferum* subsp. *somniferum* and *P*. *somniferum subsp*. *somniferum var*. *nigrum* (from now on *P*. *nigrum*) [11,32]. The *Papaver somniferum*/*setigerum* seeds reported in the archaeological record are, so far, not identified to subspecies/status level (i.e. at the wild/domesticated level) due to the lack of discriminating criteria [33,34], since all three taxa are very close in both size and shape. However, they are genetically separated (see [21]). *P*. *setigerum* is native to the Western Mediterranean area, and for this reason, it is assumed that the plant must have been first collected and cultivated in this area [11,14]. It is assumed that the wild progenitor of opium poppy would have had the same biochemical characteristics but with a lower proportion of the morphine alkaloid [35]. Therefore, Neolithic communities might have started to gather or cultivate the plant for its nutritional value, including the production of oil [36], and one could speculate that only later (yet possibly during the Neolithic), the plant could have developed higher alkaloid quantities under domestication. In Central Switzerland, at Zurich-Parkhaus Opéra, layer 13 (around 3150 cal. BC) bone tools had oil residues coming from flax and poppy, hazelnut and Brassicaceae, and it was suggested that the oil served as tool surface treatment to prolong their lifespan [37]. The state of research on poppy seed morphology does not allow us to establish any hypotheses regarding seed size and type of plant use, as it has been proven for domesticated populations of flax [38]. Since the state of the art currently suggests the use of the plant as a food and oil plant, we hypothesize that morphological changes will mostly be the result of cultivation, climatic and environmental conditions. A better reference collection must be generated in order to improve our knowledge on possible connections between poppy seed morphology and plant use.

Archaeological evidence shows that seeds of *P*. *setigerum* could have been gathered from the wild already during the Palaeolithic, as suggested in El Juyo, in Cantabria, with seeds attributed (without direct dating) to the Upper Palaeolithic [39]. Other questionable early records are reported in two Pre-Pottery Neolithic sites in Körtik Tepe, with deposits dated to 10400–9250 cal. BC [40]—but these seem to be the only finds of this plant in Turkey up to the Medieval period [41]. There are also some waterlogged finds in a PPN-well outside the Israeli coast at Atlit Yam, probably dating to 6050–5550 cal. BC [42]. However, the seeds were not directly radiocarbon-dated and could be younger intrusions as shown in e.g. [43] for cereal remains in Mesolithic layers in Europe. Most of the early evidence for poppy is actually found in Western Mediterranean Europe during the Early Neolithic. La Marmota (central Italy) is the earliest site that holds charred poppy capsules and seeds directly dated to ca. 5620–5480 cal. BC [12,44]. One charred poppy seed was found in the similarly old deposits of Peiro Signado, 5960–5720 cal. BC, in Southern France [45], but it was not directly dated. In La Draga, a pile dwelling in the northeast of the Iberian Peninsula, more than 5000 waterlogged and charred seeds were found within layer VII [46], its oldest occupation phase is dated to 5300–5150 cal. BC [47]. In the Taï cave in Southern France, 13 charred seeds of poppy were found in one pit, dated to 5270–4990 cal. BC, confirmed by direct dating of the poppy seeds [12,48]; they were found in close proximity to cereal grains suggesting an anthropogenic origin and, therefore, it is probable, that the seeds come from plants which were cultivated [48]. Other Early Neolithic sites with few confirmed poppy seeds are La Lámpara (5300–5000 cal. BC), Cueva de Los Murciélagos (5300–5000 cal. BC) and Los Castillejos (5300–5000 cal. BC) in Spain [49–52]. Direct dating of the seeds of Los Castillejos revealed that they probably belonged to younger phases documented at the site [12], which makes it clear that undated sparse finds cannot be trusted to reconstruct the history of this crop.

Charred poppy seeds were also found from early Neolithic sites (5300–4800 cal BC) in Northwestern Central Europe, in Belgium, Germany and Netherlands, belonging to the Linearbandkeramik culture (LBK: linear pottery) [12,53]. A poppy seed from Momalle’s site in Belgium is dated ca. 5300–4800 cal BC [12]. In Germany, charred seeds were also found at Vaihingen an der Enz and Nieder-Mörlen (LBK II, ca. 5350–5200 cal BC) [12,53,54] and in the Netherlands, at the site Geleen-Janskamperveld (ca. 5200–5000 cal BC) [12,55,56]. Also dated from this period, some seeds are found waterlogged in wells from Saxony, Germany [57]. The evidence of contacts between LBK and Cardial communities is not only attested in the archaeobotanical record, but also by lithic tools, ceramics and stone bracelets [58,59] and it is therefore possible that crops were also exchanged between these populations.

During the 5^th^ millennium cal BC, eastern Switzerland and the Alsace region (France) were influenced by the continental spread of domesticated plants by the LBK groups [60–63], and the southwest of Switzerland, the Valais region, was culturally influenced by Mediterranean groups [64,65]. Remarkable in this context are a few charred poppy seeds in the Alpine Rhine Valley (at Zizers, Hinkelstein group, around 4800 cal. BC [62], and Sevelen Pfäfersbüel, Roessen culture, around 4300 cal. BC [63]. While in Valais, in southern Switzerland, at the La Gillière site, dating from 4980 to 4730 cal. BC, more than 7000 charred seeds found within a hearth, pointing to an extensive use of poppy seeds [66]. In a site nearby, the Sion-Tourbillon site (Switzerland), the ceramics show similarities with the Isolino group from Northern Italy [66]. Mediterranean species at both sites suggest a Mediterranean connection [66]. These parts of Switzerland could be connected via exchanges with communities living in the French regions using the Rhone river, Lake Geneva, and other routes (even through high alpine passes) that might have existed connecting to the North of Italy or SE France [67,68].

In contrast to Early Neolithic, only very few charred remains were found in the Mediterranean area (incl. adjacent regions) in sites from the Middle and Late Neolithic (from 4500 to 2300 cal. BC). In NE Iberia, at Can Sadurní (northeast of the Iberian Peninsula), there are some undated charred seeds [69], and there is also indirect evidence of the use of poppy at the nearby site of the Gavà mines. Biochemical analysis indicating the presence of morphine on the human bone of an individual with two trepanations, as well as phytolith remains (parenchyma) from the poppy capsules in dental calculus, allegedly provide the oldest evidence of the consumption of opium poppy for sedative purposes, indirectly dated, around 3870–3365 cal. BC [70]. In SE France, seeds were preserved in waterlogged conditions, in some wells within dryland sites, at Les Bagnoles, Clos de Roque and Mas de Vignoles IX, dating between 4200 and 3090 cal. BC [71–73]. Chenet de Pierres (4338–4050 cal. BC), a dryland site located at a higher altitude (940 m asl) in the French Alps, also yielded poppy seeds alongside pottery of "VBQ" (Square Mouthed Vases) and Saint Uze ceramic styles (ca. 4500–4000 cal. BC) that attest influences from Northern Italy (in the east) and the Rhone valley (in the southwest) in the alpine regions of south-eastern France [68,74]. In Andorra, in the Pyrenees, the site of Camp del Colomer provided the evidence of opium poppy at the highest altitude (ca. 1350 m asl) in the region, associated with contexts dating ca. 4500–4000 cal. BC, and interpreted as cultivated [46].

In contrast to the Mediterranean Middle and Late Neolithic period, from 4300 cal. BC onwards, poppy is frequent in the “pile-dwelling” settlements of Northern Alpine Foreland, mostly in extremely high numbers and almost exclusively in a well preserved waterlogged state. One of the first sites to yield poppy seeds was Egolzwil 3, which also shows Mediterranean influence in objects such as flints [75,76]. Recently published investigations based on ceramic styles of Northern Alpine foreland pile dwelling sites (between 3950 and 3800 cal. BC) show for instance clear stylistic linkages between Lake Zürich and the “Néolithique Moyen Bourguignon” (namely, to the West) [77]. These linkages seem to be based on mobility of social groups [77]. This is only one example, and archaeological research on such topics is ongoing. It is highly probable that social groups not only took ceramics (or at least the ideas of ceramic shapes) with them, but also domestic plants [77]. From around 2700 cal. BC onwards, poppy becomes more rare, and from the final Neolithic Bell Beaker period (around 2400–2300 cal. BC ca) and the Early Bronze Age there are few sites with poppy and only in small quantities [31,78–82].

This state of the art opens several questions regarding the status of poppy in the Mediterranean area, its role after the Early Neolithic and whether opium poppy was already domesticated when it arrived into other areas of Europe. Another question is if it arrived there directly from the Mediterranean area. In this study we try to tackle these issues using waterlogged seeds from two areas: the western Mediterranean area and the surroundings of the Alps. However, this is a preliminary attempt in the framework of a research project that aims to understand Neolithic crop dynamics in the Northwest Mediterranean region [83]. It is intended to expand the analyses to other areas and chronologies in the near future within on-going projects devoted to the study of the domestication of opium poppy (https://opiumpoppy.hypotheses.org/). Another problem is that there are only a few sites with waterlogged poppy seeds from the Middle Neolithic and none for the Late Neolithic period in the Mediterranean area, so it is not possible to assess if the species was domesticated there or not.

This is a timely research because, first of all, a methodology to distinguish opium poppy seeds from several wild species of the genus *Papaver* has recently been established [84]. Discriminant analyses on morphological variables (shape, size and number of cells) showed rather good accuracy (over 70%) at classifying between modern species, including *P*. *somniferum* from *P*. *setigerum*. This method was also successfully applied to non-charred archaeological seeds, with the example of Zurich-Parkhaus Opéra layer 13 (Switzerland, dated to ca. cal. 3150 cal. BC [85]), where the results suggested that the domestication of the plant might be ongoing during the Late Neolithic in the area [84]. In the same study, we underlined the need for additional analyses, particularly in the Mediterranean area and of more recent sites, to reach a better understanding of the timing and geographic origin of the spread and domestication of the opium poppy in Western Europe. The recent investigation of several sites with waterlogged preservation in the Mediterranean area and south of the Alps [46,67,73,86,87] allows to make a step forward in this study that would not have been possible a few years ago.

Here, we applied the same methodology of the paper mentioned above [84] to archaeological seeds from a selection of Prehistoric sites in the NW Mediterranean and the fringes of the Alps, including the Northern Alpine foreland, in order to address three questions:

1. Is there any relation between the changes in size and shape of the seeds and their chronology? Can we detect diachronic size and shape changes?
2. Can we establish connections between archaeological seeds found in sites from the Mediterranean area and the seeds found in the Alpine area? Furthermore, what does this tell us about the process of domestication and diffusion of opium poppy outside of the Mediterranean area?
3. Can we identify domestic *Papaver somniferum* seeds in the Neolithic archaeological assemblages and in which proportion?

## Material and methods

**Fig 1:**
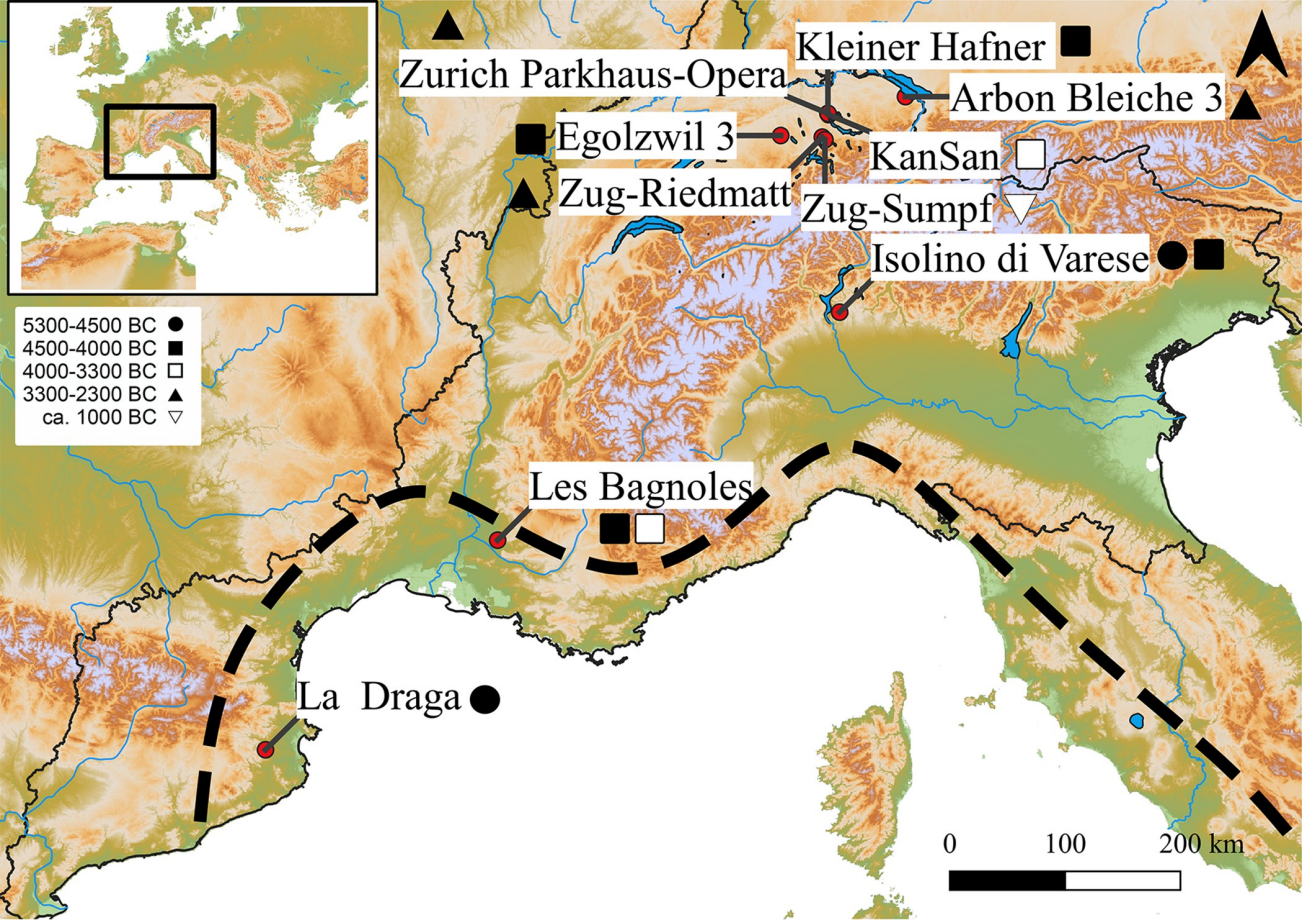
Neolithic and Bronze age sites (5300 to 1070 cal. BC) with uncharred poppy seeds analysed in this paper. Early Neolithic Sites (5300–4500 BC): La Draga and Isolino di Varese 1; Middle Neolithic 1 sites (4500–4000 BC): Egolzwil 3; Isolino di Varese 2; Zürich-Kleiner Hafner and Bagnoles 1; Middle Neolithic 2 sites (4000–3300 BC): Zürich-KanSan and Les Bagnoles 2; Late Neolithic sites (3300–2300 BC): Arbon Bleiche 3; Zug-Riedmatt, Zurich-Parkhaus Opéra; Late Bronze Age site: Zug-Sumpf. Native area of wild poppy delimited south of the dashed line. Software: QGIS3.6, © European Union, Copernicus Land Monitoring Service [2016], European Environment Agency (EEA).

A total of 295 perfectly preserved waterlogged seeds from ten archaeological sites (Table 1 and Fig 1), dating from the Early to the Late Neolithic (5150–2300 cal. BC) and one from the Late Bronze Age (1075 cal. BC), were investigated. In order to facilitate comparisons between sites, these have been grouped into six broad phases following the chronology of the AgriChange project [83]. This chronology defines the Early Neolithic (EN) from 5700 to 4500 cal. BC, Middle Neolithic (MN) from 4500 to 3300 cal. BC and Late Neolithic (LN) from 3300 to 2300 cal. BC. For the period of ca. 1050–800 cal. BC, we use the term Late Bronze Age (LBA). The Middle Neolithic period was divided into two sub-periods Middle Neolithic 1 (MN1) 4500–4000 cal. BC and Middle Neolithic 2 (MN2) 4100–3300 cal. BC. The archaeological seeds analysed in this study come from nine wetland sites and one dryland site, Les Bagnoles, however, with three wells with waterlogged deposits (Fig 1). The wetland sites are all prehistoric settlements with wooden houses on the shores of lakes or islands in lakes, known as pile-dwellings [88]. All seeds are uncharred and preserved in a waterlogged state (S1 Table). Two sites are located in the Western Mediterranean area (La Draga, Spain and Les Bagnoles, France). Isolino di Varese, Northern Italy, lies at the southern fringe of the Alps, and the remaining sites lie in the Northern alpine foreland, in current Switzerland (Fig 1).

**Table 1:** List of sites analysed in this paper.

Site	ID number	Localisation	Date (Median)	Number of seeds	Preservation	Period	Dated by	Bibliography
La Draga	80	NE Spain	5150	24	waterlogged	EN	^14^C [47]	[46]
Isolino di Varese 1	72	Northern Italy	4900	21	waterlogged	EN	^14^C [86]	[87]
Bagnoles 1, well 250	252	SE France	4150	7	waterlogged	MN1	^14^C [89]	[74]
Isolino di Varese 2	72	Northern Italy	4150	21	waterlogged	MN1	^14^C [86]	[87]
Egolzwil 3	205	Central Switzerland	4150	30	waterlogged	MN1	dendro-dated [76]	[90]
Zürich Kleiner Hafner layer 5	209	Central Switzerland	4150	30	waterlogged	MN1	dendro-dated [78]	[91]
Bagnoles 2, wells 990–994	252	SE France	3915	9	waterlogged	MN2	^14^C [89]	[74]
Zürich Kansan layer 9	220	Central Switzerland	3800	30	waterlogged	MN2	dendro-dated [92]	[93]
Arbon Bleiche 3	201	Eastern Switzerland	3375	30	waterlogged	LN	dendro-dated [94]	[95,96]
Zug Riedmatt	227	Central Switzerland	3175	30	waterlogged	LN	Typologically dated	[97]
Zurich-Parkhaus Opéra layer 13	246	Central Switzerland	3150	33	waterlogged	LN	dendro-dated [85]	[85,98]
Zug Sumpf	400	Central Switzerland	1075	30	waterlogged	LBA	dendro-dated ^14^C [99]	[100–102]

List of records analysed in this paper with their ID number, location, dates, number of seeds and preservation, organised by chronology (EN Early Neolithic; MN1 Middle Neolithic 1; Middle Neolithic 2, LN Late Neolithic and LBA–Late Bronze Age), for more details check S1 Table. ’Records’ are subsets of sites, with distinct chronological and cultural sets of data (sensu [103]). Therefore, one single site can have two records as it is the case of Les Bagnoles and Isolino.

Samples were obtained from well-dated archaeological contexts (either by dendrochronology or radiocarbon dating and in one case by typology, see Table 1) and covered different temporal, geographical and environmental conditions that could be related to poppy diffusion. This selection allowed us to assess seed size and shape changes according to time and space. For the study, we only selected perfectly preserved, uncharred (waterlogged) archaeological seeds. This type of preservation retains all seeds characteristics, such as the cells and the soft yellow parts of the hilum. Scientific plant nomenclature and classifications were discussed in the previous paper [84] and follows existing literature [11,32].

### Archaeological data collection

Only seeds identified as opium poppy, *Papaver somniferum/setigerum*, were selected. Archaeobotanists made this identification based on the appearance of cell patterns, which shows the unique characteristic feature of being areolate-reticulate compared to the other taxa present in the modern reference material. The archaeological seeds were first removed from the conserving agent (a mix of thymol, ethanol, glycerine and water), cleaned with distilled water and then partially dried with an optical cloth (Hama Lens cleaning tissue). This process was necessary to prevent light reflection due to the water and the optical cloth not leaving fibres or traces on the seeds. Finally, the partially dried seeds were easier to position allowing to take more standardised photographs and to reduce parallax error. Since the archaeological seeds were permanently stored in wet conditions we assume that no morphological changes have taken place since their recovery.

All archaeological seeds’ lateral view was photographed using a Leica Z16 APO Binocular Stereo Microscope with a digital camera Leica DFC 420 and the Leica Application Suite software (LAS 4.0, Leica^®^). The photo background and the yellow soft tissue on the hilum were removed manually using Photoshop 6 (Adobe^®^). A black mask was created using Photoshop, and the (x; y) coordinates of five landmarks [see 84] were recorded using ImageJ [104]. The seed size (length and width of the bounding box) was recorded using the rectangular tool in ImageJ. The length and width of the seeds were log-transformed [105,106]. The number of cells visible on the pictures was counted using the multi-point tool in Image J.

#### Outline analysis

The shape of all 295 archaeological seeds was described with outline analyses using elliptical Fourier transforms (EFT) [107] following the protocol developed in [84]. The (x; y) coordinates of the outline were obtained with the Momocs 1.3.0 [108] package in an R 4.0.0 environment [109]. Landmark n°2 was used as the initial point for each outline that corresponds to 360 points equally spaced points. The outlines were normalised for their position, size and orientation using full generalised Procrustes alignment of the landmarks [110]. The elliptic Fourier transform method consists of decomposing the outlines into a harmonic series of trigonometric functions, called harmonics, associated with coefficients [108], further used as quantitative shape variables.

#### Morphometrics

First, we analysed each category of morphometric descriptor separately: the overall distributions of seed lengths, widths, cell number values and shape were displayed using boxplots divided per period and Wilcoxon’s tests were performed to test for differences between periods, sites and regions. Alpha significance level was chosen to 10^−3^.

Boxplots were used to visualise differences between regions as regards the four descriptors (length, width, cell number and shape). Then, a new boxplot was plotted to visualise changes in seed length through time, with seed length of the sites in the y axis and their corresponding dates in the x axis. Finally, a chronological graph with the variations in length, width and number of cells through time was done.

**Fig 2:**
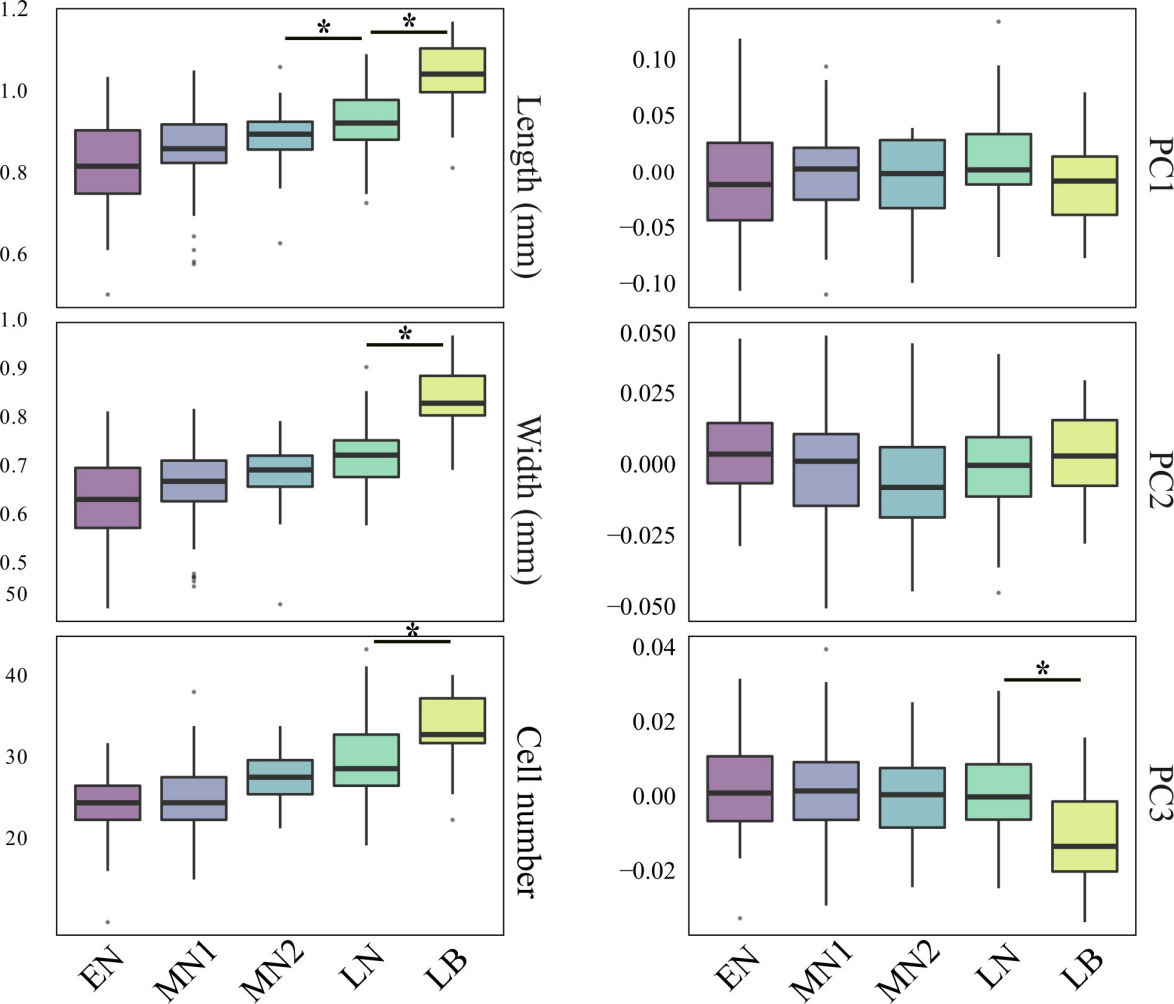
Boxplots comparing the length, width, cell number and the three first principal components (PC) of the poppy seeds shape from the chronological phases. **EN**: Early Neolithic Sites: Draga and Isolino di Varese 1; **MN1**: Middle Neolithic 1: Egolzwil 3, Isolino di Varese 2; Kleiner Hafner, layer 5; Bagnoles 1, well 250; **MN2**: Middle Neolithic 2: Zürich KanSan, layer 9, Les Bagnoles 2, well 990–990; **LN**: Late Neolithic: Arbon Bleiche 3; Zug Riedmatt, Zurich-Parkhaus Opéra, layer 13. **LB**: Late Bronze Age: the layer of Zug Sumpf. See also Fig 1 and Table 1. Pairwise differences are tested using Wilcoxon rank tests, and stars indicates P<10^−3^.

A Principal Component Analysis (PCA) was performed on the matrix of EFT coefficients to explore shape variability. Shape variability was illustrated using boxplots showing the distribution of the scores of the seeds on the first three PCA components, which together captured 90% of total shape variability. Shape differences between periods are mainly captured on PC3 (as seen in Fig 2). Therefore, the three first principal components were used in on the shape analysis.

A second PCA was used to explore the overall variability of the form (size + shape) of archaeological seeds using a combination of all morphometric descriptors (length, width, the number of cells and shape coefficients). Finally, a hierarchical clustering using UPGMA on the euclidean distance matrix between PC1:3 scores and all morphometric descriptors averaged per site is presented as an unrooted tree obtained with the package ape [111].

Each archaeological seed was classified using the linear discriminant analyses (LDA) trained on the modern material [84] and using all descriptors (length, width, number of cells and shape). To cope with the unbalanced sample size in this reference collection, we used 100 permutations [84,112], sampling in each group as much seeds as in the smallest group. For each seed, the majority classification obtained along these 100 permutations was retained. The archaeological seeds were first classified into a model including all seven *Papaver* species (LDA 1), then into a model based only on the three *P*. *somniferum* subspecies: *P*. *nigrum*, *P*. *setigerum* and *P*. *somniferum* (LDA 2), and the last LDA 3, only with *P*. *setigerum* and *P*. *somniferum*. The accuracies presented are the percentages of specimens classified into the right group. The percentages calculated for each site were represented as scatter pies on a map, to visualise spatial variation and QGIS software version 3.6 [113]. Improvements such as increasing the visibility of words and labelling were made using Inkscape [114].

### Archaeobotanical identification

A modern reference collection of 270 seeds belonging to seven *Papaver* taxa (including *P*. *dubium* L., *P*. *hybridum* L., *P*. *rhoeas* L. and *P*. *argemone* L.), with 30 seeds measured per taxon (for details, see [84]), was used to identify the archaeological seeds using a “predictive” linear discriminant analysis. For each seed, the dominant classification obtained along the 100 permutations was considered as the predicted class. All descriptors (length, width, shape and number of cells) were used, following [84].

## Results

### Results per period

The distributions of length, width, cell numbers, and shape (first 3 Principal Components (PC) representing 90% of variance) values show a slow change from the Early to the Late Neolithic, and a larger change to the Late Bronze (Fig 2). Wilcoxon rank tests indicate that seed assemblages of different periods usually differ from one another regarding length and width, with the few exceptions of neighbouring periods such as no differences (P>10^−3^), between the seeds of the Early Neolithic and Middle Neolithic 1, and Middle Neolithic 1 and Middle Neolithic 2 (S2 Table). Differences, P<10^−3^, in the number of cells mainly concern Middle Neolithic 2, Late Neolithic and Late Bronze periods. Changes in shape are smaller and only significant, P<10^−3^, between Late Bronze Age and all the Neolithic assemblages on PC3 (Fig 2 and S2 Table).

**Fig 3:**
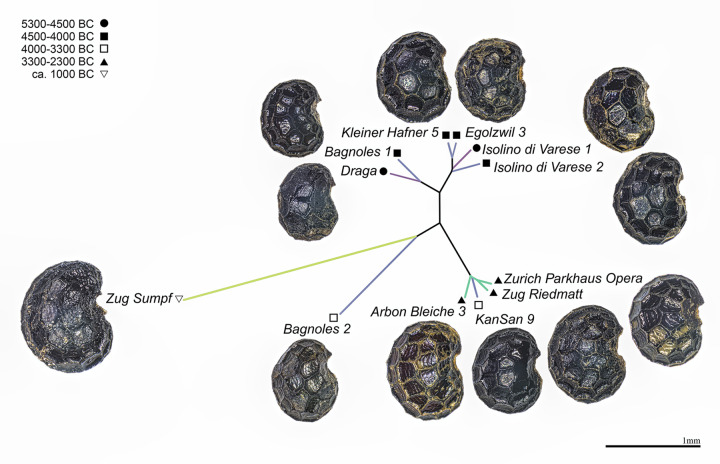
The unrooted tree obtained with hierarchical clustering on the euclidean distance matrix between Fourier coefficients, length, width and number of cells per site. The colours of the lines represent the period colours seen in the previous figures. Photos by Raül Soteras. For details to the sites see Table 1.

The hierarchical clustering based on all descriptors (Fig 3) allows to identify five clusters. The Mediterranean sites of Les Bagnoles 1 and La Draga form one cluster. The sites from the South and North of the Alps from the Early Neolithic and Middle Neolithic 1 cluster together (Isolino 1 and 2, Egolzwil 3 and Kleiner Hafner 5), while the three sites belonging to the Late Neolithic (Arbon Bleiche 3, Zug Riedmatt and Zurich-Parkhaus Opéra) and one site from Middle Neolithic 2 (Zürich KanSan 9) compose the third cluster. Late Bronze Age Zug Sumpf is isolated and distinguished from all the others. The same occurs to Les Bagnoles 2 (the younger well) that is isolated from the other seeds from Middle Neolithic 2 and Late Neolithic.

### Results per region

**Fig 4:**
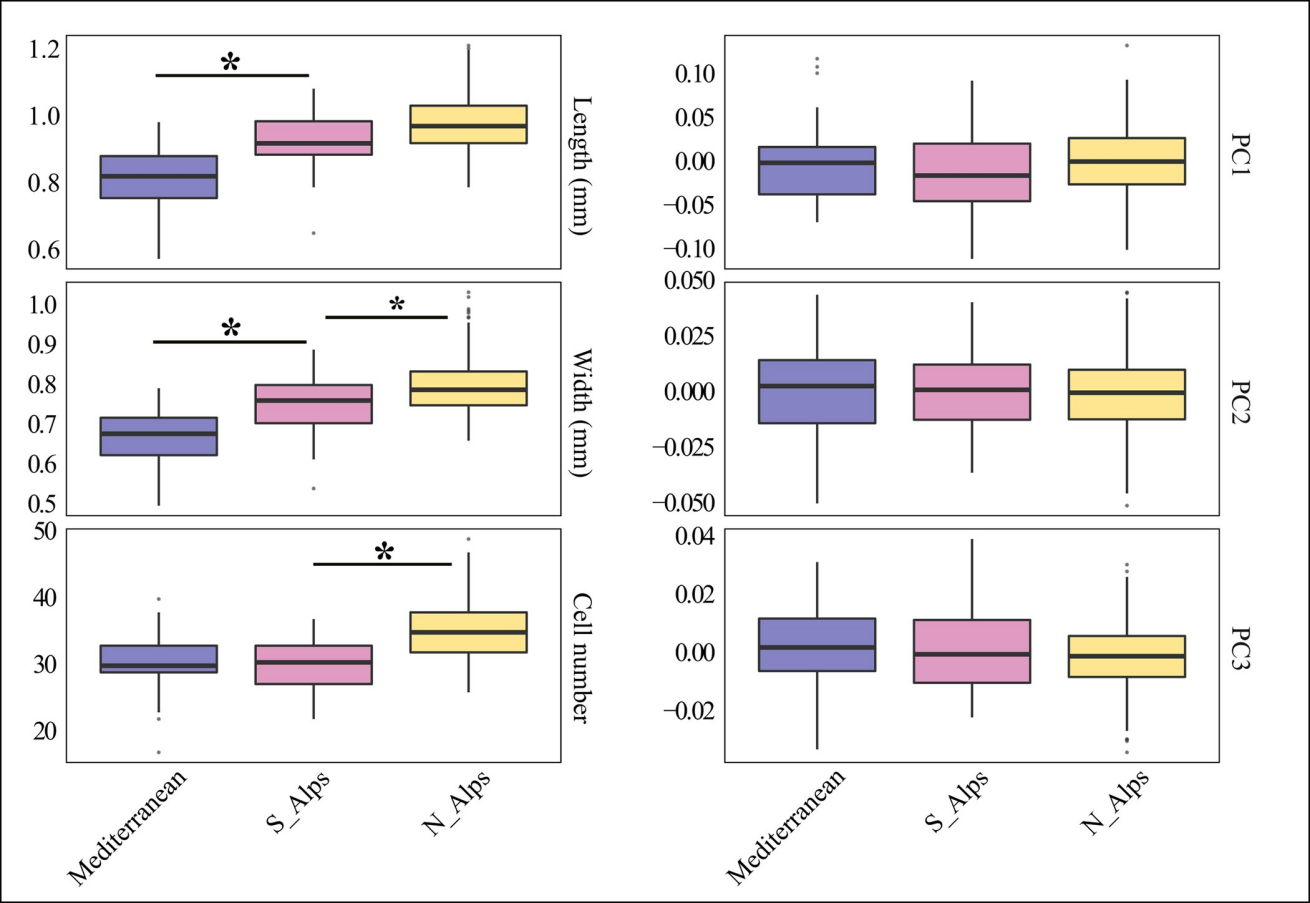
Box plots comparing the length, width, cell number and the three components of the three first PC scores of the outline shape analysis of the poppy seeds from different regions. Mediterranean region boxplot includes La Draga and Les Bagnoles 1 and 2; South of the Alps boxplot includes Isolino 1 and Isolino 2, and North of the Alps has all Swiss sites. Pairwise differences are tested using Wilcoxon rank tests, and stars indicate P<10^−3^.

Seeds also differ between regions (Fig 4), with the length and width of assemblages from the Mediterranean being smaller than in the other regions, as demonstrated by the Wilcoxon tests. The Wilcoxon tests (S2 Table) show statistical differences in length and width, P<10^−3^, between the Mediterranean region and the other regions North and South of the Alps despite no difference in terms of the number of cells and seed shape (P>10^−3^), as well as, between the Mediterranean region (La Draga + Les Bagnoles) and the South of the Alps region (Isolino). In terms of width and cell number, the assemblages from the South are different (P<10^−3^) from the seeds of the North of the Alps. A clear distinction for the North of the Alps is observed in the number of cells, which is larger in this region (Fig 3). In terms of shape, there is no significant change in the different regions.

### Evolution of seed length per settlement phase through time and regions

**Fig 5:**
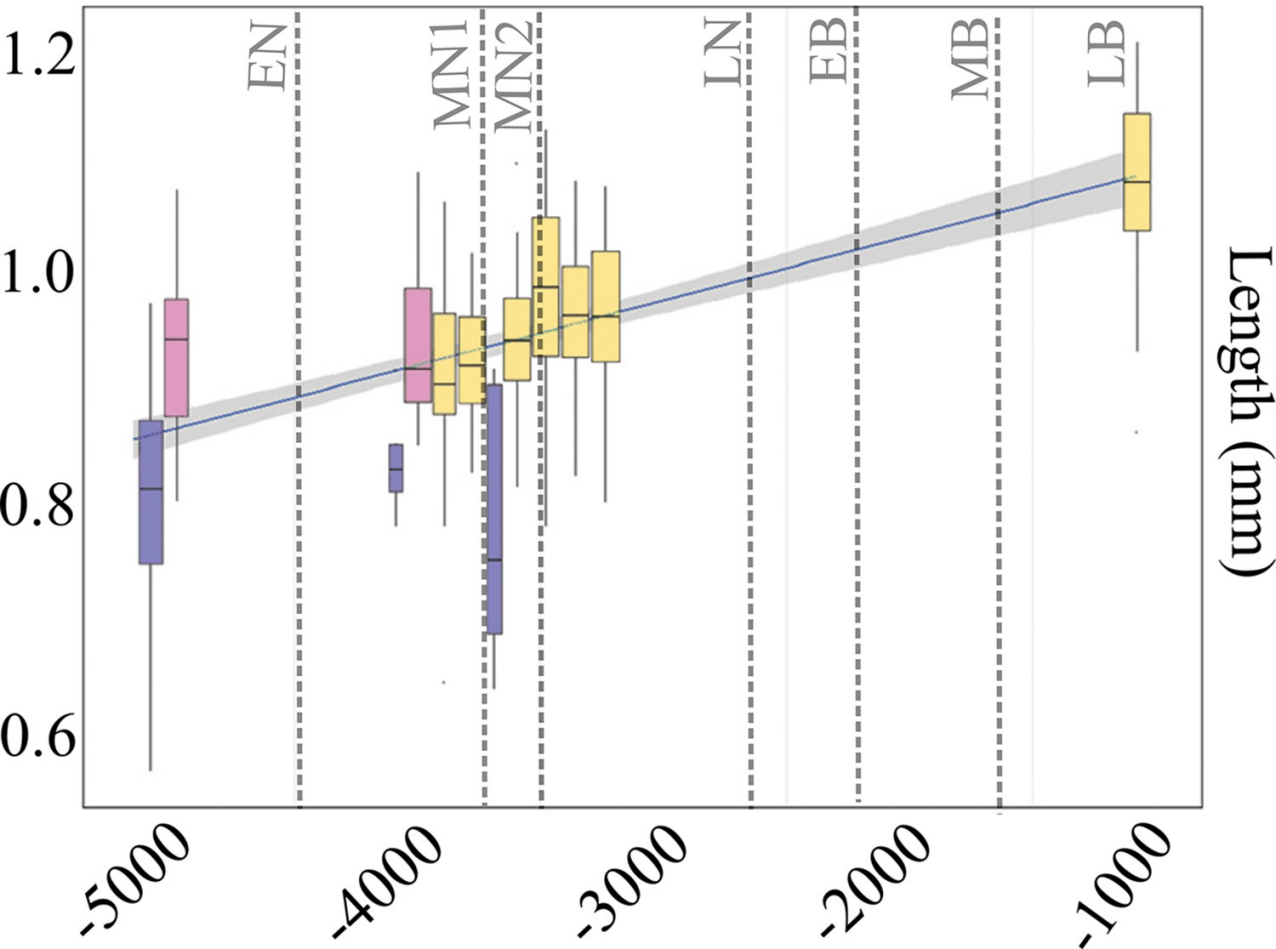
Boxplots with the length values per site, the average date in the continuous x-axis and regression lines. Ordered by a period as in Table 1 with all the sites analysed here, the colours are the same as in the previous regional graphs.

Seed length is often used as a parameter to compare overall seed size changes diachronically. We addressed this comparison at a settlement phase level per site and classified sites per period phases in order to observe possibly different dynamics between regions (Fig 5). The seeds from the North of the Alps sites show a continued increase in length from the Middle Neolithic 2 to the Late Neolithic. Within the 1000 years between the Late Neolithic (ca. 3150 cal. BC) and the Late Bronze age (ca 1075 cal. BC), a more significant increase in length is evident, as shown in Figs 5 and S1.

However, when combining all the criteria, the estimated increase of change in the seeds (p<10–15, adj. r2 = 0.344) and the pace is 0.057 mm in length,0.049 mm in width and 0.26 in the number of cells per millennium. (S3 Table).

### Assignation of archaeological seeds

Even though all archaeological seeds included in this study belong morphologically to the *P*. *somniferum* group, we used our modern reference collection from different *Papaver* species, and a predictive linear discriminant analysis to identify the archaeological seeds. LDA 1 assigned only four seeds wrongly (S2 Fig). Three seeds were attributed to *P*. *dubium* and one seed to *P*. *hybridum*. This result demonstrates the agreement between both methods (S2 Fig).

**Fig 6:**
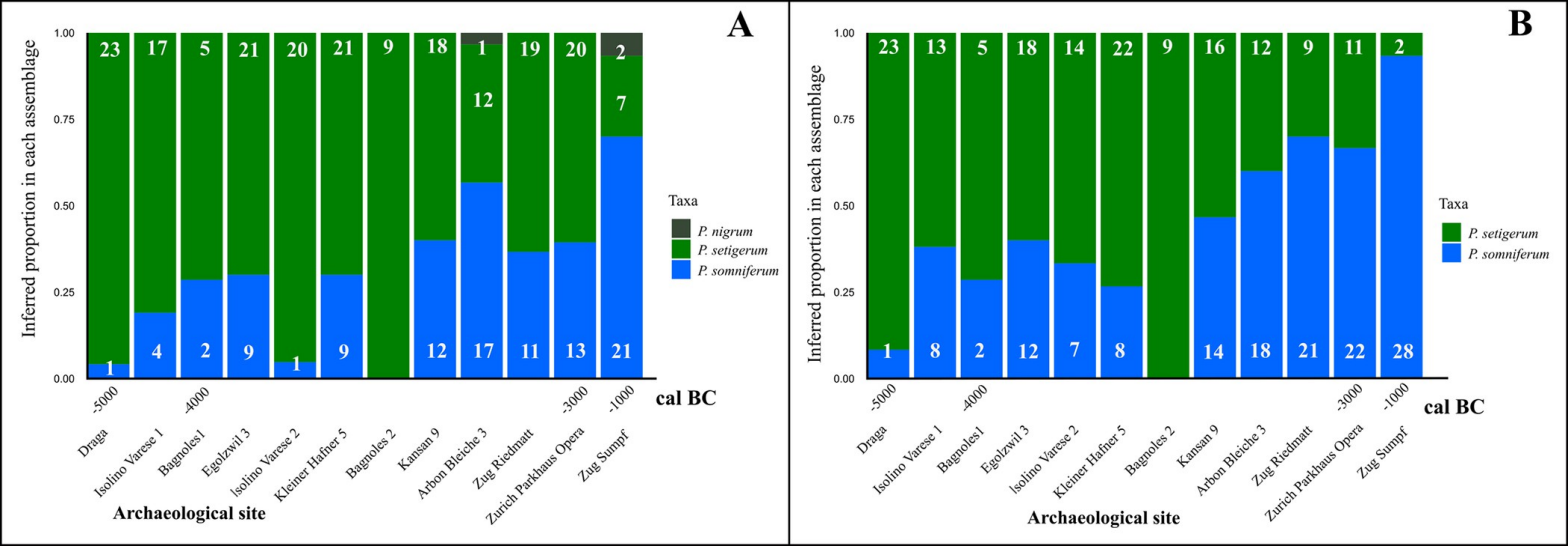
Assignment of archaeological seeds to modern *P*. *somniferum* morphotypes. **(A)** LDA 2 trained on the *P*. *somniferum* group (*P*. *setigerum*, *P*. *somniferum* and *P*. *nigrum*) using all descriptors. (**B)** LDA 3 trained on the *P*. *somniferum* group (*P*. *setigerum*, *P*. *somniferum*) using all descriptors. The number of seeds attributed is listed on each represented colour.

Archaeological seeds were assigned with the same methodology to modern species only belonging to the *P*. *somniferum* group (LDA 2, Fig 6A). Most of the seeds were assigned to *P*. *somniferum* and *P*. *setigerum*. The number of seeds allocated to *P*. *nigrum*, only five, is insignificant compared to the other two taxa. Only two seeds of Arbon Bleiche 3 and three seeds from Zug Sumpf were identified as the modern species of *P*. *nigrum* (Fig 6A). Therefore, another LDA was performed only on the two *P*. *somniferum* subspecies, *P*. *setigerum* and *P*. *somniferum*. The results of LDA 3 changed slightly. The main pattern is an overall increase in the proportion of seeds assigned to *P*. *somniferum* (Fig 6B), especially in Isolino Varese 2. The seeds from La Draga and Les Bagnoles 2 are the most similar to *P*. *setigerum*. The Late Bonze age site, Zug Sumpf, is the only site where most seeds were assigned to modern *P*. *somniferum*.

#### Geographical morphotype of poppy assemblages

**Fig 7:**
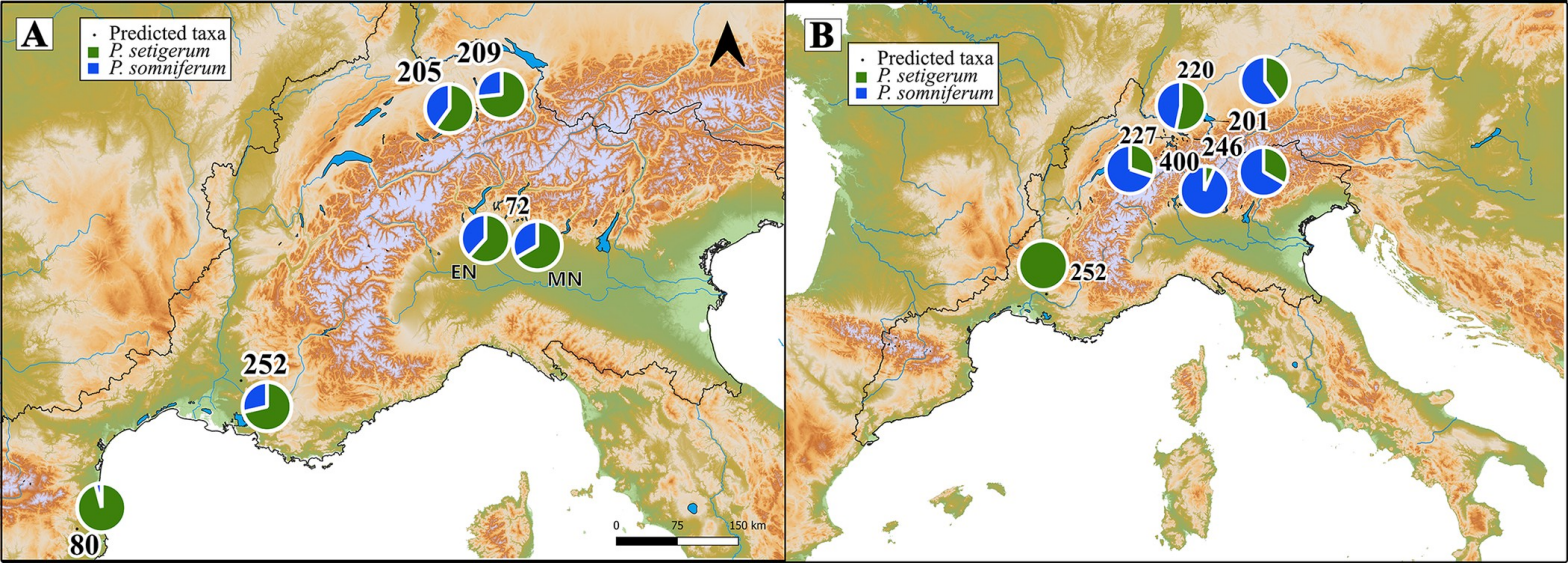
Map of the sites showing the proportion of seeds assigned to *P*. *setigerum* (green) and *P*. *somniferum* (blue) by LDA3. (A) Assemblages from the Early Neolithic and Middle Neolithic 1 period (5700–4000 cal. BC). **EN**: 80) La Draga, 72) Isolino di Varese 1. **MN1**: 72) Isolino di Varese 2; 205) Egolzwil 3, 209) Zürich Kleiner Hafner layer 5, 252) Bagnoles 1, well 250. (B) Assemblages from the Middle Neolithic 2, Late Neolithic and Bronze Age (4000–800 cal. BC). **MN2**: 252) Bagnoles 2, well 990–994, 220) Zürich Kansan layer 9; **LN**: 201 Arbon Bleiche 3, 227) Zug Riedmatt; 246) Zurich-Parkhaus Opéra layer 13 and **LBA**: 400) Zug Sumpf. (Software: QGIS3.6, © European Union, Copernicus Land Monitoring Service [2016], European Environment Agency (EEA).

During the Early and Middle Neolithic 1 periods (5700–4000 cal. BC), all sites show most poppy seeds with traits similar to *P*. *setigerum*, especially La Draga and Les Bagnoles 1 and 2 in the Mediterranean area (Fig 7A). In the sites from the other regions, North and South of the Alps, the proportion of seeds with *P*. *somniferum* traits is higher. For the Middle Neolithic 2, the only site in the Mediterranean area, Les Bagnoles 2 (the younger wells), had all nine poppy seeds assigned to *P*. *setigerum* (Fig 7B). At younger sites located in Switzerland, a larger proportion of seeds is allocated to *P*. *somniferum*. The proportion of *P*. *somniferum* in this area reaches its maximum, especially in Late Bronze Age Zug-Sumpf, where most seeds are attributed to this taxon (Fig 7A).

## Discussion

This paper shows the application of four descriptors: length, width, number of cells, and shape, to study poppy seeds’ variability from archaeological sites dating between 5150 and 1075 cal. BC from the Mediterranean and North of the Alps regions. The morphometric studies compared archaeological seeds with modern references, assigned archaeological seeds to *P*. *setigerum* and *P*. *somniferum* and highlighted chronological and geographical changes in seed morphology.

An initial question of the study was to understand the relation between the changes in size and shape of the seeds and their chronology, and this study found that the seeds show a slow continuous change from the Early to the Late Neolithic, and a larger change to the Late Bronze age (Figs 2 and 5). The Wilcoxon tests (S2 Table) show no statistical differences in length and cell number between the early periods. In contrast, differences in width and shape are only visible on the seeds from the Late Bronze age compared with earlier periods. These results support the idea that Middle and Late Neolithic seeds outside the natural range of *P*. *setigerum* come from plants which were in the process of domestication, whereas the LBA seeds possibly come from domesticated plants. With the available data we cannot establish when this full-domestication happened and if there was an introduction of new forms from an unknown area at some point during the Bronze Age. It is actually known that during the Bronze Age there were many introductions of new taxa, mainly from the (far) east [115,116]. Nevertheless, the fact that some changes are observed locally in the Late Neolithic leaves the door open to a local domestication process that should be further explored in the future.

The second question in this study sought to determine possible connections between archaeological seeds found in sites in the Mediterranean area and the seeds found in the Alpine area. The most obvious finding to emerge from the analysis is that the seeds from the Mediterranean region are smaller than those found in the other regions, as confirmed by the computation of Wilcoxon rank tests (Fig 4 - P<10^−3^). These Wilcoxon tests on length and width measurements indicate that the seeds from the southern fringes of the Alps (Isolino site) are different from those of the Mediterranean region (La Draga + Les Bagnoles) but similar to those north of the Alps. This result shows that the seeds had already slightly changed their morphology compared with seeds from the native area of the wild progenitor, even already in early times (Early and Middle Neolithic). This happened obviously relatively quickly when the species was brought to outside of its native area (Mediterranean). Connections between Isolino and the earliest pile dwellings north of the Alps have already been postulated based on other archaeobotanical evidence [86]. It would be interesting to know if such changes would be visible also in more northern lying early Neolithic contexts such as the LBK-wells in Saxony with waterlogged preservation [57] as the domestication of the poppy plant could have also occurred in other areas such as Belgium and Netherlands (see e.g. [117]), where early finds have been dated [12].

When comparing the different areas, width and cell number of the seeds from the North of the Alps are higher than those of the seeds from the South of the Alps and the Mediterranean (Fig 4). This result suggests that the seeds from the North of the Alps differ from those within the native area. The plants from which these seeds come might be more advanced in the domestication process compared to the plants from the Mediterranean. However, these results may be somewhat limited by two aspects: the number of specimens when comparing regions and, more importantly, the absence of seeds from the Mediterranean from later periods when trying to understand the process of domestication and diffusion of opium poppy outside the Mediterranean area.

The first refers to the fact that the number of seeds used is not the same for each region, as there are more seeds from the Alpine area than from the Mediterranean. This is due to the type of preservation since only uncharred (waterlogged) seeds can be studied with our method (but see below). In the Mediterranean area there are only very few examples of pile dwellings and dryland sites with structures that could contain this type of preservation, for example, wells. The number of seeds per region is 40 from the Mediterranean area (3 records/2 sites), the South of the Alps (2 records/1 site) has 42 seeds, and the North of the Alps (7 records/site) has 213 seeds.

The other limitation is that no poppy seeds from the native area dating from the Late Neolithic and Bronze age have been recovered yet. Therefore, a direct assessment and comparison between seeds found in the native area and the seeds in the surroundings of the Alps is challenging. Nevertheless, the main results are that the seeds found in the native area (Mediterranean) are always smaller than those from Early Neolithic and Middle Neolithic sites in other areas. In contrast, the specimens out of the native area are larger and tend to increase from the Early Neolithic up to the Late Bronze age. However, as noted before there are gaps of hundreds of years and a linear “evolution” of the seed size cannot be proven.

One final aspect to consider is environmental factors. The Circum-Alpine area is significantly wetter than the Mediterranean area. Therefore, it cannot be excluded that seed size may be affected to some extent by the changing growing conditions. This aspect will need to be further investigated when the dataset is improved, and seeds from other climatic and environmental regions can be included in the analysis.

### Identification of *P*. *somniferum* in the investigated sites and the role of poppy

The third question in this research was to assess if the seeds found at these sites were morphologically similar to the domestic species, *P*. *somniferum*. When allocating archaeological seeds to modern species of opium poppy by LDA, the seeds from the native area were mostly assigned to the wild morphotype, *P*. *setigerum*. In contrast, the proportion of seeds assigned to the domestic type is generally higher in the other regions. This proportion varies in terms of period, and the highest value is obtained for the seeds from the Late Bronze Age (Figs 6 and 7).

The seeds from La Draga (ca. 5300–5150 cal. BC) in NE-Iberia are mainly allocated to the wild form: the seeds are smaller and have fewer cells than modern *P*. *somniferum*. The taxa spectrum at the site is dominated by cultivars such as naked wheat and barley, poppy was identified as a cultivar based on its density of finds and its ubiquity [46]. Our results indicate therefore a sort of “pre-domestication cultivation” [118] of poppy, because the seeds are morphologically wild.

In Les Bagnoles, a Middle Neolithic site located in Southern France, in the southern Rhone valley (Fig 1) and dating to ca. 4250–3800 cal. BC, the taxa spectrum shows a dominance of naked wheat and barley for the Middle Neolithic 1 and for the Middle Neolithic 2 barley and naked wheat with an increase of glume wheats. Flax is present in all phases, while the representation of pulses is variable, mostly dominated by pea [73]. Poppy seeds are frequent in the Middle Neolithic 1 and fewer in the Middle Neolithic 2.

These seeds are assigned to *P*. *setigerum*, too. Especially the nine seeds from the Middle Neolithic 2 have similarities in terms of length, width and number of cells with the seeds of La Draga. In comparison, the seven seeds of Middle Neolithic 1 are larger, and some are allocated to *P*. *somniferum* (Figs 6 and S2). Large quantities of poppy seeds were mainly found in the oldest well 250 (ca. 4250–4050 cal. BC), some in a younger well 990 (dating to 4050–3980 cal. BC) and a few in the youngest (3940–3780 cal. BC) well 994 suggesting cultivation of poppy at the site throughout the Middle Neolithic period [73]. The seeds in this site were not well preserved despite being waterlogged, and only 16 seeds could be measured. This relatively small sample size should be extended before exploring more in detail the differences between the two Middle Neolithic phases.

Isolino Virginia, a pile dwelling site on an island in lake Varese, is located in Northern Italy, South of the Alps (Fig 1), and has two phases of occupation, one around 4950–4700 cal. BC and one around 4250–3650 cal. BC [86]. The archaeobotanical research is still ongoing [87] but preliminary data revealed that naked wheat and naked barley dominate as crops in the first phase, while a more diverse crop spectrum is found in the second phase, also involving the presence of glume wheats. Flax is present in both phases, as well as pea [87]. The assemblage is different from the glume wheat-dominated assemblages found in other North-eastern Italian sites [119]. Poppy has been known since the first occupation phase at Isolino and appeared in large numbers [120]. For both occupations, 21 seeds were analysed, among which eight were allocated to *P*. *somniferum* for the Early Neolithic period layer and seven from the Middle Neolithic layer, corresponding to less than 40% of the seeds. One possible interpretation of this result is that the domestication of the plant is ongoing, possibly due to the long period of cultivation of over 500 years (since poppy was first found at the Early Neolithic site of La Marmotta, central Italy) but also due to the location of the site, which lies outside of the natural area of distribution of wild opium poppy. The Isolino finds are the earliest poppy seeds showing some domestication traits known today.

In the seven sites North of the Alps (for dating see Table 1), poppy seeds are numerous and are generally better preserved than in the other sites, particularly in comparison to La Draga and Les Bagnoles. This exceptional preservation is related to taphonomic processes in lake environments in the circumalpine area, where organic deposits are often found with excellent preservation conditions [8,121]. On the other hand, the large number of seeds means that more well-preserved remains were available. The seeds are typically found in organic detritus layers [121] accumulated under or around the (mostly stilted) houses [122,123]; in two sites, Zurich AKAD-Seehofstrasse [78] and Zug-Sumpf [124] accumulations of seeds were found within ceramic pots suggesting their storage and culinary use. High densities of several thousand poppy seeds per litre of sediment are found in several pile-dwellings, with extreme numbers mainly in sites with “Horgen” style ceramics (around 3300–2900 cal. BC), so e.g. in Zurich-Parkhaus Opera, Horgen-Scheller 3, Zürich-Kansan and Pfäffikon-Burg [85].

During the Middle Neolithic 1 (around 4200 cal. BC), opium poppy was brought to the Swiss Plateau and cultivated by the Swiss pile-dwelling communities with “Egolzwil” ceramic style along the same crops that were grown in Isolino, namely naked wheat (mostly tetraploid) and multi-rowed barley, few flax finds and several remains of pulses, especially pea [78,125,126]. The evidence of cultivation is first based on the large quantities of seeds in multiple sites in the Swiss Plateau [31,78,80–82] and secondly on the lack of the wild ancestor in the area. The LDA 3 model (Fig 6B) allocated the poppies of the Middle Neolithic period (4500 to 3300 cal. BC) mainly to *P*. *setigerum*; however, the proportions are higher in Middle Neolithic 1 (over 60% in Egolzwil 3 and layer 5 of, Zürich-Kleiner Hafner). For Middle Neolithic 2 (Zürich-Kansan), more than 50% are allocated to the domestic type (Figs 6 and 7). This result may be explained by the fact that Egolzwil 3, Zürich Kleiner Hafner layer 5 and Zürich Kansan layer 9 sites belong probably to the earliest (archaeobotanically investigated) examples of the spread of poppy into the Swiss Plateau, which might have been brought from the NW Mediterranean, as also many archaeological findings show (see above).

In a diachronic perspective, more details are observable. Around the end of Middle Neolithic 2 and during Late Neolithic (from 3000–2300 cal BC, Arbon Bleiche 3, Zug Riedmatt and Zurich-Parkhaus Opéra “Horgen” layers), 60 to 70% of the seeds are inferred to be *P*. *somniferum* species. A comparison of these findings with previous morphometric results [84] confirms that during the Late Neolithic, poppy seeds are becoming larger and more similar to the morphotype of the domestic type. The large numbers of seeds, by far over 1000 poppy seeds per litre of sediment [27,67], found in the waterlogged archaeological sites reflects the importance of this crop. The main cereals during this period were emmer, tetraploid naked wheat and a multi-rowed barley plus high amounts of flax [80,82].

The Late Bronze age seeds from one site–Zug Sumpf–are 93% allocated to the *P*. *somniferum* species according to the LDA 3. This suggests that poppy plants were fully domesticated from the morphometrical point of view provided by the seeds. The main cereals during this period are multi-rowed barley, spelt and emmer wheat, and additionally there are high amounts of broomcorn and foxtail millet and faba bean [127].

Our results suggest that opium poppy cultivation started in the Mediterranean area with morphologically wild forms. Seed morphology only changed very progressively until we find fully domesticated morphotypes in Switzerland during the Late Bronze Age. Part of this process took place–and possibly accelerated–outside the distribution area of the putative wild ancestor, *P*. *setigerum*. In Switzerland, the seeds gradually and systematically increased in size until the Late Neolithic. The most significant change is observed in the Late Bronze Age but we cannot clearly establish where and when this change first originated. These findings hence do not assert that it was a strictly a local evolution of the crop. The lack of sufficient appropriate contexts in the Mediterranean area dating to the Late Neolithic, and the lack of available waterlogged remains from the investigated region dating to the Bronze Age also does not allow to exclude the possibility that the domestication process also included these areas. The whole process possibly involved repeated exchanges and inter-breeding of genetic stock or cultivated varieties between the Alps and the Mediterranean and maybe even other areas during a very long period of time. This is also shown by the considerable genetic variability of *P*. *somniferum* as shown by Hong et al. [21]. Yet unresolved is the fact that *P*. *setigerum* and *P*. *somniferum* show considerable genetic differences (most *setigerum* is tetraploid, whereas *somniferum* is diploid), therefore more research is needed in order to understand the story of the domestication of opium poppy.

It is therefore clear that this study cannot establish with certainty the domestication centre of opium poppy. For this, more research in other European areas and additional sites is needed, i.e. studying other sites with waterlogged deposits such as the above-mentioned Early Neolithic LBK wells in Saxony, Germany [57] or the recently investigated pile-dwellings in the Central Balkans [128]. Another important prerequisite for the further investigation of this question would be to develop an additional methodology for charred remains which then would allow to trace a more comprehensive investigation of poppy domestication. Adding charred poppy remains is important for future research since the methodology used in this paper was tested only on waterlogged material [84]. Carbonisation experiments could allow to assess the possibility of classifying charred seeds. However, prior studies noted the strong effect of charring on seed shape and cells pattern [129]. Creating a new method for charred poppy seeds would maximise the chances of understanding the history of the crop by increasing the number of sites available to be studied, as there are many dryland sites with charred preservation than waterlogged sites. In addition, more details of the phylogenetic and phylogeography of opium poppy from modern and ancient DNA would help track its origin(s) and dispersal.

## Conclusions

In this paper, we show the application of four descriptor types (length, width, number of cells and shape) to study the variability of waterlogged archaeological poppy seeds from ten archaeological sites dating from the Early to the Late Neolithic and one site from the Late Bronze age (5150–2300 cal. BC and 1075 cal. BC). Our study clearly demonstrates that wild poppy started to be cultivated alongside domestic crops such as barley and naked wheat or pulses in the western Mediterranean, and this set of crops then spread to regions North of the Alps. In the surroundings of the Alps, where the majority of our data come from, we observed an ongoing domestication process that was morphologically complete about 4000 years later. However, information from the latest Neolithic periods (Corded Ware, Bell Beaker) and the Early and Middle periods of the Bronze age from our study area and from other areas is missing, so we cannot verify continuity between the Late Neolithic and the Late Bronze Age, although the available information would make this a plausible scenario.

The findings from this study make several contributions to the current knowledge. Firstly, by the accomplishment of distinguishing archaeological seeds of opium poppy using the four descriptors. Secondly, it is the first study that provides evidence of poppy domestication, which, as the results suggest, probably happened beyond the Mediterranean area. It is difficult to know if the plant spread only as a crop or also as a weed, but there is certainty that the plant was cultivated in the Mediterranean area, as shown by the large stores of seeds, and hence it is more likely for the plant to have spread from the Mediterranean regions into the north of the Alps as a cultivar.

It was not possible to assess charred remains of opium poppy and some research gaps that need to be addressed in the near future were identified. Consequently, more work needs to be done to validate if and when domestication also occurred in the Mediterranean, or if this is a result of separating wild poppy from its native area / natural habitat. In this aspect it would be interesting to integrate Early Neolithic (LBK) poppy seeds from wells with waterlogged preservation but also more seeds dating to the Middle to Late Neolithic periods and the Bronze age from Mediterranean sites. The latter will require new research, since we do not know of any finds of additional waterlogged poppy seeds in the area dating to this period.

In order to continue the research presented in this paper, we aim to continue developing the model with modern reference material from different regions (at best in combination with genetic data) and to expand the archaeological material to sites with waterlogged deposits in central, eastern and southern-eastern (Balkans) Europe.

The observations that have been possible for opium poppy in this paper, namely, that the domestication process is visible and traceable outside of the area of spread of the wild ancestor of the crop, might be relevant for the study of other domestication processes during the Neolithic (and later) periods.

## Supporting information

S1 Fig Boxplots with the values of length, width and cell number per site. Box plots with the average date in the continuous x-axis and regression lines. Ordered by period as in Table 1, colours are the same as the period ones shown previously.
(TIF)

S2 Fig Assignment of archaeological seeds to modern *Papaver* seeds morphotypes LDA 1 trained on all seven *Papaver* species using all descriptors. The number of seeds attributed is listed on each represented colour.
(TIF)

S1 Table List of archaeobotanical information for each poppy seed. (XLSX)

S2 Table Wilcoxon tests to assess differences between periods, sites and regions. (XLSX)

S3 Table Table with the estimation of pace of change of the different descriptors. Estimation of the pace of change in length, width and number of cells per millennia.
(DOCX)
