# Supplementary figures and images for: Morphometrics of waterlogged archaeological seeds give new insights into the domestication and spread of Papaver somniferum L. in Western Europe
Source: PLoS One. 2023 May 25;18(5):e0286190. doi: 10.1371/journal.pone.0286190 (PMC10212148; doi:10.1371/journal.pone.0286190)

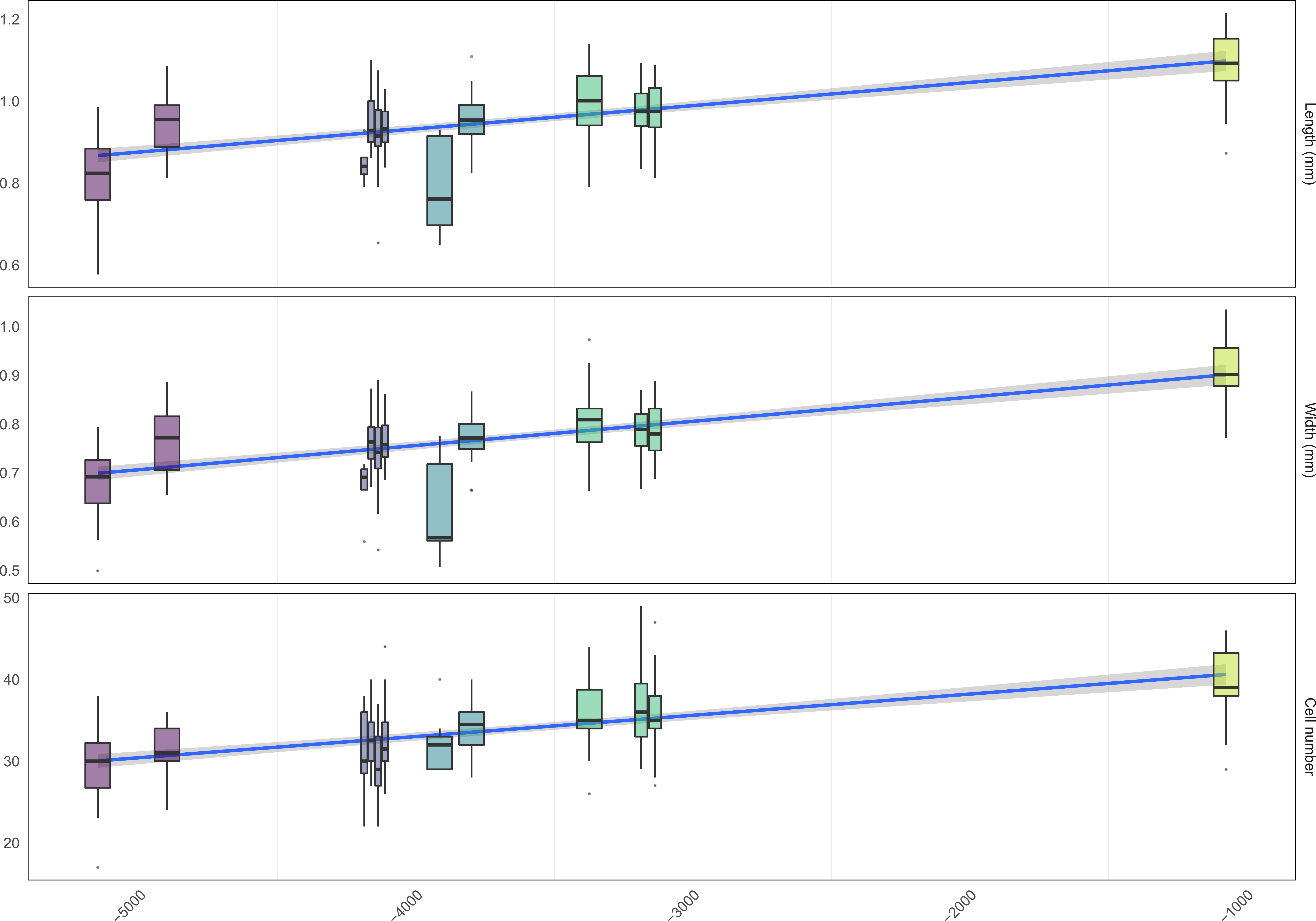

Supplement: S1 Fig — Box plots with the average date in the continuous x-axis and regression lines. Ordered by period as in Table 1, colours are the same as the period ones shown previously. (TIF) [file pone.0286190.s001.tif]

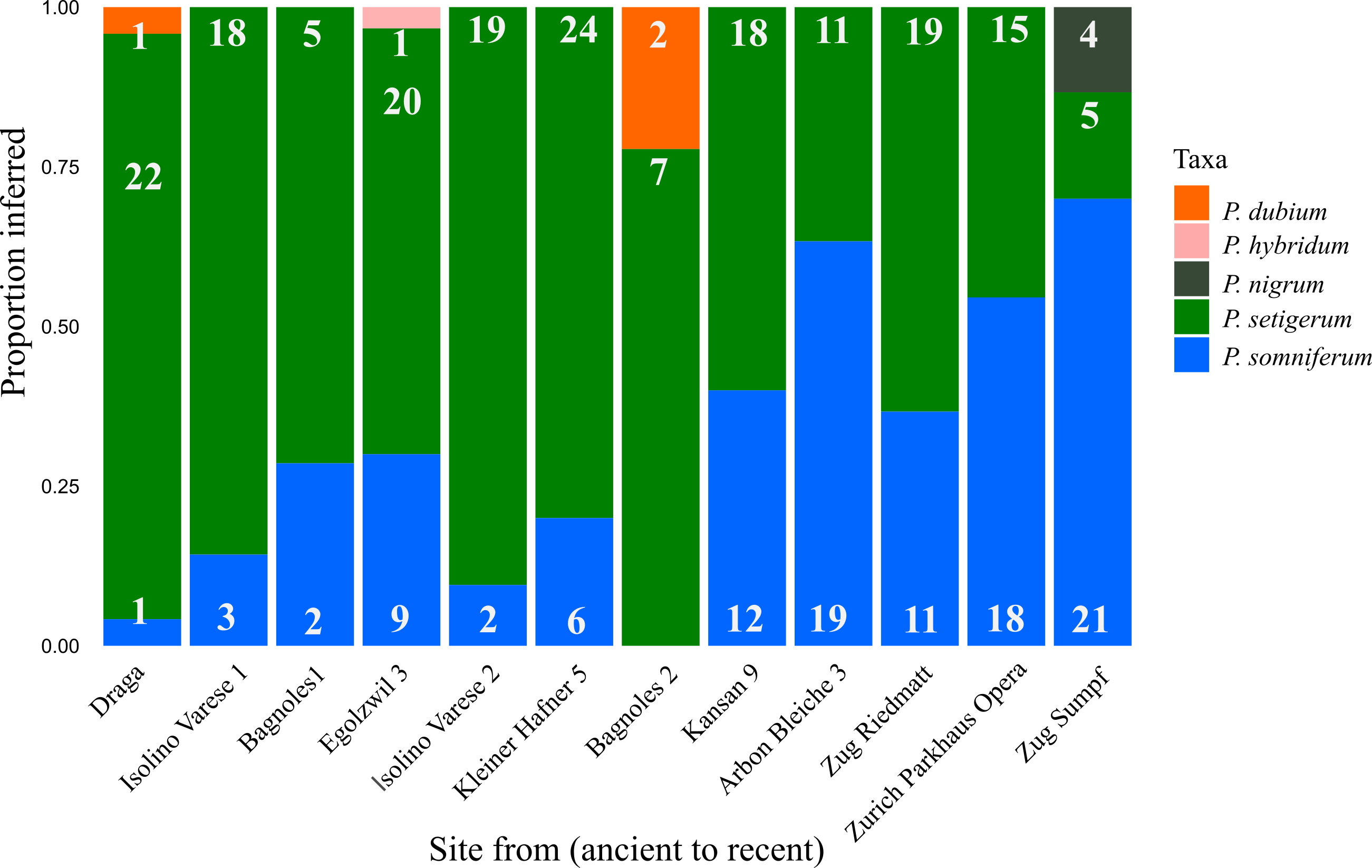

Supplement: S2 Fig — The number of seeds attributed is listed on each represented colour. (TIF) [file pone.0286190.s002.tif]
