# Supplementary material for: Morphometrics of waterlogged archaeological seeds give new insights into the domestication and spread of Papaver somniferum L. in Western Europe
Source: PLoS One. 2023 May 25;18(5):e0286190. doi: 10.1371/journal.pone.0286190 (PMC10212148; doi:10.1371/journal.pone.0286190)
Supplement: S3 Table — Estimation of the pace of change in length, width and number of cells per millennia. (DOCX) [file pone.0286190.s005.docx]

**S3 Table. Table with the estimation of pace of change of the different descriptors.**

| **term** | **estimate** | **std.error** | **statistic** | **p.value** | **Descriptor** |
| --- | --- | --- | --- | --- | --- |
| 1 (Intercept) | 1.16 | 0.0172 | 67.2 | 3.84e-180 | Length (mm) |
| 2 date | -0.0000567 | 0.00000455 | -12.5 | 7.24e- 29 | Length (mm) |
| 3 (Intercept) | 0.955 | 0.0144 | 66.4 | 1.14e-178 | Width (mm) |
| 4 date | -0.0000496 | 0.00000379 | -13.1 | 4.56e- 31 | Width (mm) |
| 5 (Intercept) | 43.4 | 0.877 | 49.5 | 2.13e-144 | Cell number |
| 6 date | -0.00260 | 0.000231 | -11.2 | 1.48e- 24 | Cell number |

Table with the estimation of the pace of change in length, width and number of cells per millennia
